# Radical Cure of Hernia

**Published:** 1883-05

**Authors:** 


### Selections.

### Radical Cure of Hernia.

In two clinical lectures in the *Indian Medical Gazette*, Dr. R. McLeod describes the method of operating for the radical cure of hernia, which he has practiced in several cases during the last two years, and gives a table of the cases with details of the results. His plan of operation — not peculiar to himself — is to isolate the sac quite up to the internal ring, there place a catgut ligature around it moderately tight, and tie the same thread at intervals of half an inch lower down the sac three times more. He cuts off the sac below the lowest ligature. With a Wood's needle he then stitches the inner pillar and conjoined tendon to the outer pillar and Poupart's ligament, leaving the ligatured neck of the sac as a plug in the inguinal canal. The wound treatment is antiseptic, and Dr. McLeod lays great stress on this point, insisting that the operation is not justifiable unless this precaution is taken. In all, Dr. McLeod has performed this operation twenty-eight times with a total mortality of six. Of these cases seven were patients with strangulation of the hernia, and this operation was done as a sequel to ordinary kelotomy; of these one died of collapse eight hours after the operation, and another ten hours after the operation of laparatomy, performed five days and a half after the first operation on account of persistence of the symptoms of obstruction. In four patients the hernia complicated large scrotal tumors requiring operation. Two of these patients died from septicæmia, but in neither of them was there any peritonitis or mischief in the seat of the operation upon the hernia. The remaining seventeen were cases of reducible hernia, and among them there were two deaths — one from pyæmia, and one patient who was removed from the hospital ten days after the the operation, died two days later, probably from carbolic acid poisoning. These cases do not show the operation to be one attended with serious risk if performed antiseptically. Four of the six fatal cases, in two the operation cannot be credited with causing the mortal shock. In the two cases fatal from septicæmia, the poison appears to have been absorbed from the scrotal, and not from the inguinal, wound. The other two deaths are directly traceable to the operation. Evidently it is not a procedure to be entered upon lightly, or without clearly explaining the risks to the patient. But whenever kelotomy has to be performed, it would appear to be wise if possible to suppliment it by an attempt to procure a permanent cure of the affection, for this adds nothing to the danger of the original step, and indeed by closing the track into the peritoneal cavity possibly lessens one of its perils.

